# The information gain of explicitly provided over self-generated contextual knowledge for behavioral control

**DOI:** 10.1371/journal.pone.0318994

**Published:** 2025-02-07

**Authors:** Lukas Magnaguagno, Stephan Zahno, Ernst-Joachim Hossner

**Affiliations:** Institute of Sport Science, University of Bern, Bern, Switzerland; Opole University of Technology: Politechnika Opolska, POLAND

## Abstract

In time-pressured decisions, humans exploit contextual knowledge to reduce uncertainty about the unfolding situation and to improve behavioral control. However, in complex real-world settings, it remains unclear whether the explicit provision of contextual information is beneficial or not. We thus examined the information gain of explicitly provided information as a function of expertise, information uncertainty and acquisition phase. To this end, we measured the positioning of female handball players (*N* = 36 experts + 36 near-experts) in a virtual-reality defensive task as a function of their teammates’ defensive-strength patterns, which was either explicitly instructed or had to be self-generated. Furthermore, the certainty of provided information was experimentally varied (67% vs. 83% consistent information). All eight groups–expertise (2) x acquisition condition (2) x information certainty (2)–improved performance in terms of the positional difference in their defense movements, meaning that they either moved more sideways to support their neighboring teammate or remained more often in their position when no support was required. However, an explicit-knowledge test showed no differences regarding pattern detection between the acquisition conditions, implying that the performance enhancement of the self-generated groups was not due to explicit-knowledge accumulation. Most notably, experts generally benefitted from explicit instructions whereas for near-experts, an information gain could only be revealed for comparably certain information. This interaction implies that future research on explicit provision vs. self-generation of contextual knowledge should pursue a more differential approach, thereby also considering gender and age as well as personality factors.

## Introduction

Behavioral control in complex decisions involves acquiring and integrating information from diverse input variables [1]. The richer the information and the greater the number of action options, the more the behavior is afflicted with uncertainty [2]. Since decisions under uncertainty are related to situational probabilities, uncertainty can be reduced by gathering available information from two broad sources; current sensory information and prior contextual information [3, 4]. Contextual information is of particular relevance in sport games; wherein the detection of regularities refers to probability relationships between a large number of situations and decisions have to be made under high time pressure [4, 5]. However, this kind of information on regularities can also be provided by coaches. Imagine, for instance, you are the coach of a tennis player who is going to play his next game against Rafael Nadal. You know that Nadal serves with a high probability down the T-line on deuce court [6] and that such knowledge about an opponents’ preferences can boost your player’s performance [7], particularly when sensory information is not available or not reliable [8–10]. Would you decide to provide this contextual information to your player explicitly, or would you let your player accumulate this information through his own experience so that he self-generates contextual knowledge for upcoming situations? And on what factors would your decision depend?

At this point, the potential benefits of implicit vs. explicit learning are evident. Implicit knowledge can be defined as knowledge that is difficult, if not impossible, to verbalize [11]. This particularly refers to the acquisition of unconscious knowledge of rule-governed information without awareness of what is being learned [12]. Contrastingly, explicit learning involves full or at least partial awareness of the learned information and can thus be defined as information that can be consciously accessed and verbally communicated [13]. Assuming that learning processes are not simply dichotomous [14], explicit learning has been further differentiated in regards to rule-application and rule-discovery. In rule-application, the nature of the probability relationship is explicitly instructed. A rule-discovery approach would only provide information about the existence of probability relationships among situational features, such that the specific relationship has to be discovered [15].

Typically, sport game studies on the accumulation of contextual information examine players’ performance in cognitive aspects such as decision making, or anticipation based either on implicitly or explicitly acquired knowledge. On the one hand, findings showed that players who gathered the contextual information implicitly or through an explicit rule-discovery approach increased their performance accuracy [10, 16, 17], particularly for highly skilled players [10, 16–21] and for action-congruent situations [18–20] and even more pronounced for the combination of both factors [19, 20]. On the other hand, the explicit provision of contextual information was shown to have a positive effect on performance as well [21–24]. Again, highly skilled players outperformed their counterparts [25], particularly in action-congruent situations [24, 25].

In contrast to studies focusing only on one of the two acquisition types, research comparing the utilization of implicit versus explicit knowledge is rare in the literature on performance enhancement and can mainly be found in studies on motor learning. However, regarding the effects of contextual information on motor control, empirical evidence on the use of specific information is not only limited but also rather inconsistent [15, 26–31]. Whilst Shea et al. [28] claimed that withholding the information is more beneficial than providing it, other research groups did not find any differences between implicit and explicit learning [29–31]. Consequently, further investigation is warranted to understand the conditions under which implicit and explicit–possibly self-generated–knowledge of contextual information influences motor performance as well as decision making.

In this context, three factors that seem to crucially affect the efficacy of both implicit and explicit learning are separately examined so far. First, in the field of motor control, previous research explored the (un)certainty of the provided information by comparing different levels of (un)certainty. In summary, these studies have shown that, when the information exceeds a certainty threshold (for 50%, see [30]; for 75%, see [15, 26, 27]), an implicit acquisition regime improves motor learning to a greater extent than providing instructions on a rule-application basis. Second, the level of expertise might be an additional factor that affects the benefits of implicitly acquired and explicitly provided contextual information as mainly investigated in studies on players’ performance in more cognitive tasks [10, 16, 17, 19–21, 25]. Third, in both research fields, task load seems to be another moderating factor on the integration of contextual information meaning that learners suffer more from explicit information in complex tasks; as it is argued that the high attentional demands leave only limited resources for the processing of contextual information [4, 24, 28]. However, there is a lack of research thus far regarding the utility of instructions about contextual information compared to withholding this information in such complex, real-world scenarios, whilst simultaneously considering varying information certainties, the learners’ domain-specific expertise, and different phases of the learning process.

To the best of our knowledge, only two studies can be found that considered the consequences of the last-made statement, i.e., real-world scenarios of behavioral control in terms of players’ performance, different acquisition types, varying information certainties, learners’ domain-specific expertise, as well as different acquisition phases. This at least partly applies to the study conducted by Gredin et al. [8] who examined female soccer players’ ability to anticipate the direction of an oncoming opponent’s action. Results showed that explicit compared to implicit contextual information was beneficial only in situations of highly certain contextual information. This effect appeared to be of relevance in the presence of highly reliable kinematic information. However, whilst Gredin et al. [8] examined performance enhancement with and without the provision of contextual information as well as under different degrees of (un)certainty of the contextual information, the learners’ domain-specific expertise and different phases of the acquisition process were not part of their investigation.

In contrast, a recent study by Magnaguagno et al. [32] not only assessed the role of explicit information provision vs. self-generated contextual information of different degrees of (un)certainty, namely in handball players’ behavioral control in a complex virtual-reality environment, but considered the additional above-mentioned factors (i.e., expertise, information certainty, and acquisition phase) as well. The defensive behavior of the players was investigated as a function of contextual knowledge about the teammates’ defensive strengths in 1-on-1 situations. In handball defense, players often face the decision of moving sideways to support their neighboring teammate or holding their central position to defend their direct opponent. When the teammate is strong and likely to win the 1-on-1 situation, the player should remain in their position. In contrast, when the teammate is weak and likely to lose the 1-on-1 situation, moving towards him/her to stop the attacker is the correct decision as the attacker with the ball is currently a bigger threat in this game situation. In the experiment, the contextual knowledge about the teammates’ strength was either self-generated by own experiences or explicitly provided by the experimenter. To assess the extent to which the explicit provision of more or less valid information influenced players’ performance, the authors first calculated a positional-difference score. High scores reflect a more pronounced supporting behavior towards a (generally) weak teammate than towards a (generally) strong teammate. The mean explicit information gain was then estimated based on (in)congruency effects. More precisely, the difference between the behavioral marker of contextual-information exploitation (i.e., positional difference) of the explicitly instructed groups and the self-generated groups was used to estimate the information gain of explicitly provided contextual knowledge for different degrees of (un)certainty. Furthermore, these estimations were made separately for learners of different expertise levels and also under consideration of the acquisition phase (i.e., amount of specifically accumulated prior knowledge). On the basis of these estimations, Magnaguagno et al. [32] concluded that players with less expertise should generally benefit from the provision of explicit information over the entire acquisition phase; especially when the certainty of the information is high. In contrast, higher level players’ gain from explicitly provided information seems to be limited to an early acquisition (i.e., initial phase), as well as to situations under higher certainty. For these expert players, the provision of information with lower certainty could have a negative impact on performance; particularly in cases when self-generated knowledge could not yet be sufficiently established. Notably, these findings are also supported by other research, namely by partially observable Markov decision process simulations which showed that incongruent information negatively affects anticipation only in the beginning, and of lesser skilled players [5].

In our eyes, this interpretation significantly extends the existing theoretical understanding of the issue of providing learners with explicit knowledge by proposing a testable information-gain function considering the interaction of the level of expertise, information (un)certainty and the acquisition phase. While the model is based on data from sport-specific situations, we would argue that, in principle, this approach should be applicable to behavioral control in complex decision tasks across domains of expertise.

However, the conclusions of Magnaguagno et al. [32] were derived from estimations. Thus, further research is required to explicitly test the model and to disentangle the effects of multiple conditions under which self-generated and explicitly provided knowledge of contextual information affects behavioral control in applied settings. To this end, the present study examined the information gain of explicitly provided information as a function of expertise level, information (un)certainty, and acquisition phase. More precisely, we reapplied the immersive environment of the handball set-up used by Magnaguagno et al. [32] and compared expert and near-experts female handball players, who were either explicitly provided with contextual knowledge or received no instructions, in two conditions of information certainty (67% vs. 83%) and over two different acquisition phases (initial vs. final).

In doing so, our experimental design tested the model proposed by Magnaguagno et al. [32] in terms of context (i.e., learner’s level of expertise, information (un)certainty, amount of accumulated self-generated prior knowledge) that determines whether or not explicit contextual information should be provided to learners. In addition, the current study sought to extend the existing theoretical understanding on the benefits of explicitly provided information by assessing the research question in an applied setting. On the one hand, this topic has previously been addressed foremost in basic research, and on the other hand, findings might be of high potential relevance for coaching practice in sport games as game analytics could be done with or without a specific focus on contextual information (e.g., opponent’s behavior).

Building on previous research cited above, we specifically expected that experts outperform near-experts regarding the strength-depended positional difference and that both expert groups improve their defense performance over time [32]. Furthermore, we hypothesized that the information gain from the explicit provision of contextual knowledge is more pronounced for higher certain information [30, 32] but negative effects were expected when situation-specific knowledge could not be sufficiently established by own experience (i.e., early acquisition phase) [15, 26, 32]. More precisely with respect to the interaction of the three factors (i.e., expertise, information (un)certainty, and acquisition phase), we expected that near-experts benefit generally more from explicit information provision than experts; particularly when the accumulation of self-generated prior knowledge is advanced (i.e., late acquisition phase) and the certainty of the information is high [32]. In contrast to near-experts, we hypothesized that experts’ information gain increases under high certainty but especially in the early acquisition phase. Additionally, we predicted a detrimental effect of lower certainty information on the information gain of experts [32]. Besides, as a standard measurement in studies including an implicit or self-generated group, we assessed the extent to which the participants were aware of the experimentally induced pattern. In this regard, we expected that pattern detection rates would be related to the acquisition condition (self-generated vs. explicit) and information (un)certainty (67% vs. 83%); such that the explicit groups would outperform the self-generated groups [31] and the higher-certainty groups would outperform the lower-certainty groups [32]. Finally, we tested for demographic differences between the eight experimentally sub-groups and hypothesized for the interaction of the three factors (i.e., expertise, acquisition condition, and information certainty) that experts have more practice hours and game competitions compared to near-experts.

## Materials and methods

### Ethic statement

The ethics committee of the Faculty of Human Science at the University of Bern approved the study protocol prior to recruitment (approval number: 2020-11-00003). The recruitment period for the study started at 2021-09-20 and ended at 2022-01-06. Written informed consent was obtained by all participants prior to participation.

### Participants

A total of 36 expert (*M*_age_ = 21.94 years, *SD* = 3.55 years) and 36 near-expert (*M*_age_ = 25.61 years, *SD* = 6.62 years) female handball players participated. Female handball players alone were recruited because participants with high expertise were needed and the entirety of adult and youth male handball players in Switzerland, where the study was conducted, had already been invited to participate in previous experiments on contextual knowledge in 1-on-1 handball defense to our lab.

An a priori statistical power analysis using MorePower 6.0.4 [33], with a significance level of α = .05 and a power of [1 − *β*] = .80 indicated that a sample of 80 participants was necessary to detect a theoretical minimum effect size (η_p_^2^ = 0.10) of interest. To calculate the practical effect size is an appropriate strategy for applied studies without the possibility to firmly estimate the effect size from relevant prior literature due, for instance, the novelty of the topic (for example, see [34]). The a priori analysis was performed for a three-way ANOVA design on expertise (2) x information certainty (2) x acquisition phase (2) with repeated measures on the last factor. Unfortunately, the sample size was restricted by the underlying population of professional female handball players in Switzerland and their availability. Eventually, we were able to recruit 36 expert (approximately 25% of the population)–instead of 40 –and 36 near-expert female handball players.

The expert players had 12.64 years (*SD* = 3.34 years) of practice experience with an average of 9.76 hours (*SD* = 2.43 hours) of practice per week. These experts had 9.86 seasons (*SD* = 3.12 seasons) of competition experience, with 26.78 games (*SD* = 5.37 games) per season. They played in the highest national division of Switzerland. The 36 near-expert players had 12.21 years (*SD* = 7.15 years) of practice experience with an average of 4.28 hours (*SD* = 1.48 hours) of practice per week. These near-expert players had 10.14 seasons (*SD* = 5.90 seasons) of competition experience with 17.61 games (*SD* = 6.75 games) per season. They played in the fourth or fifth national division and had no experience at a higher level.

The participants of both expertise groups were assigned to one of four experimental conditions. These sub-groups differed by the acquisition condition (self-generated knowledge vs. explicit provision of pattern information) as well the certainty of the contextual information (67% vs. 83% trials validly complying with the underlying rule of the teammates’ defensive strength). Whilst the *explicit groups* were informed of the pattern of their teammates’ defensive strengths in advance and repeatedly over the acquisition phases, the *self-generated groups* had to generate this information by themselves. The certainty of the contextual information consisted of two different distributions; such that the rule on the teammates’ defensive strengths was implemented in either 67% or 83% of the trials. Consequently, each participant (either expert or near-expert) took part in a single acquisition and (un)certainty condition.

The assignment to the experimental sub-groups was conducted separately for the expert and near-expert players in a quasi-randomized fashion. Two factors were considered: team membership and real-world defensive decision-making quality. Whilst the information about players’ current teams could be gathered through the recruitment process, the decision-making quality, in accordance with the experimental task, was rated by the players’ coaches on a 4-point scale. Coaches were particularly instructed to rate the player’s defense quality in situations in which they would need to decide whether to move sideways to support their neighboring teammate or to remain in their central position, since no support movement is required. Subsequently, these two factors built the basis for the quasi-random grouping so that–separately for both expertise levels–each of the four experimental groups contained an equal number of participants recruited from the partaking teams as well as participants with comparable decision-making qualities. Accordingly, the averaged decision-making quality showed no main effect for the four experimental sub-groups (experts: *p* = .989, near-experts: *p* = .953).

To further check the sub-group allocation procedure, five separate three-way ANOVAs expertise (2) x acquisition condition (2) x information certainty (2) were conducted to examine potential differences in relevant demographic characteristics. As expected, expert players practiced more hours per week, *F*(1, 64) = 134.30, *p* < .001, η_p_^2^ = .68, and partook in more games per season, *F*(1, 64) = 39.50, *p* = .001, η_p_^2^ = .38, than their near-expert counterparts. Furthermore, near-expert players were older than expert players, *F*(1, 64) = 8.20, *p* = .006, η_p_^2^ = .11. In contrast, neither a main nor any interaction effect was revealed for years of practice experience (*p*s ≥ .197) and seasons of competition experience (*p*s ≥ .313), respectively. Hence, we would like to claim that our allocation procedure successfully produced balanced groups; meaning that–in the expertise-related internal comparison–the four sub-groups initially neither differed in decision-making quality nor in any potentially relevant demographic characteristics.

### Task and apparatus

**Fig 1:**
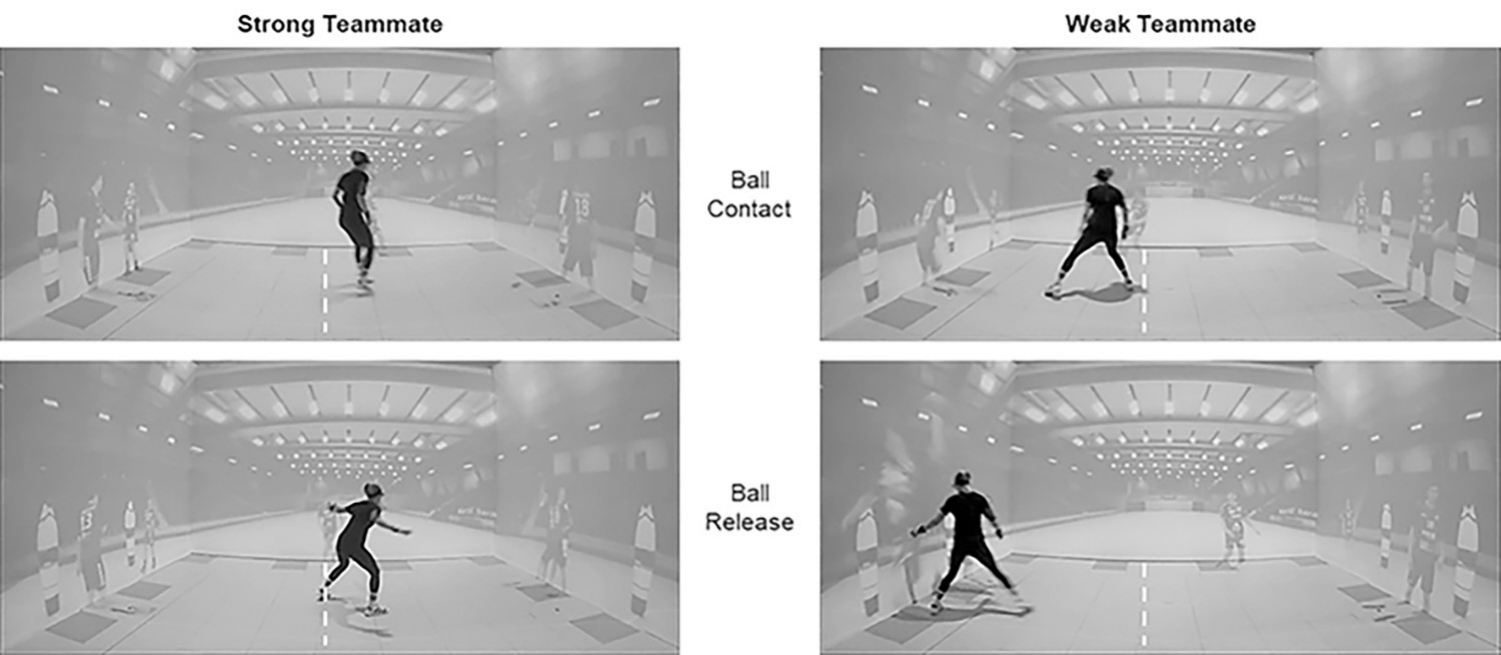
Video scenes presented in the CAVE and exemplary test stimuli (1:1 towards the throwing-arm side) with either a strong teammate (left column) or a weak teammate (right column) at ball contact (top row) and ball release (bottom row), with ball release at the moment of either the teammate’s foul or the attacker’s throw. The dotted white line on the floor–marked here for illustration–indicates the origin for the calculation of the positional-difference variable. Please note that the projection of the spherical 360° movie was centered at the participant’s starting position, such that the participant’s perspective almost perfectly matched the real-world defense situation.

Participants watched real-world video clips of dynamic 3-on-3 handball situations from the perspective of the central defense player. These situations depicted–due to availability from previous studies–the central defensive player’s left and right male teammates as well as three male offensive players. The clips were captured from a tripod 1.70 m above the ground at the position of the central defender with a GoPro Omni 360° camera at 60 Hz. Subsequently, the video footage was rendered into a spherical 360° movie (Autopano Video Pro, Kolor SAS, France) and further edited by extracting unwanted noise and by removing the visible tripod with a fitting environment (Adobe Premiere Pro CC, Adobe Systems Software, Ireland). To achieve a high ecological validity, the clips were presented in a life-sized virtual-reality environment using a custom CAVE system consisting of six cluster workstations and 11 projectors (Barco F50, 2560x1600, 60 Hz). The video footage was projected on a 6.00 m x 3.75 m front wall, two 11.00 m x 3.75 m sidewalls and the 6.00 m x 11.00 m floor. In relation to these lab dimensions, the participants’ starting position on the 6 m line of the handball field was 3 m from the front wall as well as from both sidewalls such that the front wall corresponded to the 9 m line of the handball field. The participants’ perspectives were thus almost perfectly matched to real-world defensive situations (see Fig 1; for a video, see SwitchTube: https://tube.switch.ch/videos/dZMIDhdB3n). Each clip started with a pass from the center back, followed by three prelude passes before the final offensive action. The video stopped 2.0 s after this final action. Additionally, 1.5 s freeze frames were added at the beginning and at the end of each clip, resulting in overall clip durations of about 8 s to 12 s. Participants were instructed to act in response to the video clips of the real handball players, as they would in a real-world environment, with the core objective to prevent goals.

While participants watched the video clips and acted as the central defender, data on their defensive movements were collected by a motion-capture system at 200 Hz (Optitrack; Naturalpoint Inc., Corvallis, OR). The setup consisted of 18 cameras (Prime 13W and 17W) that were placed above and all around the projected court. Participants wore a headband and a backstrap, each affixed with four marker cluster, such that participants’ head and trunk movements could be tracked by the passive marker system. The marker clusters were then each averaged to calculate the two-dimensional trajectories (x: left/right, y: backward/forward) in Matlab 2016a (The Mathworks Inc., Natick, MA).

### Test stimuli

After using expert ratings to select the most realistic scenes from the originally recorded footage, a total of 132 video clips were included in the study. These clips were subdivided into 84 experimental and 48 additional scenes. The 84 experimental scenes ended with a 1-on-1 situation (body feint towards the side of the throwing-arm) between one of the participant’s teammates and the respective left or right offensive back. In these situations, the defensive quality of the teammate who was responsible for defending the attacker’s final action was systematically varied to be either weak or strong. Specifically, one of the two teammates was the weak defender, who usually lost the 1-on-1 situations causing the scene to end with a throw at the goal. Relating to the manipulated certainty of contextual information, the distinct distributions equated to the weak defender losing these situations in either 67% or 83% of cases whilst in the remaining scenes (33% and 17%, respectively), the weak defender won the 1-on-1 situations by stopping the attacker with a foul, according to the rules. Consequently, for the weak teammate, the losing situations can be identified as pattern-consistent clips, whilst the winning situations qualify as pattern-inconsistent clips. For the strong defender, the distributions were vice versa. By presenting the clips with such frequencies, the predominant defensive patterns could be inferred by the participants. These distributions are relevant to the player in the central defense position, since she needs to anticipate whether to move sideways to support the teammate in order to prevent a throw, or to remain in her central position in foul situations, when no support movement is required. Accordingly, more sideways movements are indicated for the weak teammate, who loses in 67%/83% of cases, and fewer sideway movements are indicated for the strong teammate, who wins in 67%/83% of cases.

In half of the 84 experimental scenes, the weak defender was positioned on the left and the strong defender on the right side, and vice versa for the other half of the scenes. The weak and the strong teammate were always impersonated by the same player; meaning that, for example, the player who acted as the weak defender on the left side continued to defend weakly in the subsequent block of trials after having changed position to the right side. Within the 42 scenes per side, half of the attacks were performed against the weak defender and the other half against the strong defender. In order to create a more realistic variance within the presented defensive situations, the 84 experimental video clips were complemented by a total of 48 additional scenes in which no particular defensive patterns were enacted; neither regarding the teammates’ defensive qualities nor the outcome of the final offensive action.

In order to compile the test videos, the clips were organized into 6 blocks of 32 clips each. In each block, 24 clips were experimental clips and 8 clips were additional scenes that were included to increase situational variance (for an overview of the experimental design, see S1 Table). In both distributions (i.e., 67% and 83% groups), the same 12 experimental clips were presented in all 6 blocks; namely, 2 pattern-consistent and 1 pattern-inconsistent clips defended on each side (left and right) for the weak as well as strong teammate (= 3 x 2 x 2). These clips were complemented by 12 further experimental scenes. In the 67%groups, these scenes were split into 8 pattern-consistent clips with two clips of the weak and strong teammate on both the left and right side, and 4 pattern-inconsistent clips of the weak and strong teammates on both the left and right side. In contrast, all of the 12 further experimental scenes in the 83% groups were pattern-consistent with three clips of the weak and strong teammates defending on both the left and right sides. In total, for the 67% groups, this procedure resulted in 16 pattern-consistent clips (= 2 x 2 x 2 + 8) and 8 pattern-inconsistent clips (= 1 x 2 x 2 + 4); for a distribution of 16/24 pattern-consistent clips. For the 83% group, this procedure resulted in 20 pattern-consistent clips (= 2 x 2 x 2 + 12) and 4 (= 1 x 2 x 2 + 0) pattern-inconsistent clips; for a distribution of 20/24 pattern-consistent clips.

The order of the 32 trials per block was quasi-randomized. For half of each experimental group, the weak teammate defended on the left side for the first 16 clips and on the right side for the last 16 clips, with the strong teammate positioned accordingly. For the other half of the group, the players started on opposite sides (i.e., weak teammate on the right and strong teammate on the left). In each half of a block, the presentation of pattern-consistent and pattern-inconsistent clips reflected the induced distribution conditions. After the first 16 clips of each block, a 10 s break was inserted in the compiled video to allow participants to orient themselves with respect to the switched positions of their teammates.

For fair comparisons, analyses were solely based on the trials of the 12 constant experimental clips per block that, to recall, included a similar distribution of 8 pattern-consistent and 4 pattern-inconsistent clips in both groups. Additionally, to increase reliability of the measures and to assess performance changes related to the acquisition of prior knowledge, the experimental trials from the beginning (i.e., block 1 and 2) and the end of the experiment (i.e., block 5 and 6) were conflated. In this way, each 16 pattern-consistent and 8 pattern-inconsistent trials were available to calculate dependent measures for the phase in which prior knowledge could not be sufficiently established by own experience (i.e., early acquisition phase) and for the phase in which the accumulation of self-generated prior knowledge is advanced (i.e., late acquisition phase). Notably, as the participants’ performance was only analyzed in relation to the constant experimental clips, potential differences from the early to the late acquisition phase cannot be attributed to differences in the presented clips.

### Procedure

Participants were tested individually in single sessions of approximately 75 min. After providing informed consent, participants were instructed on the task and equipped with the marker-affixed bands for motion tracking. Subsequently, participants were allowed to accustom themselves to the experimental setup over 4 familiarization trials. After clarifying any possible questions, participants completed 6 experimental blocks with 90-s breaks in between.

To provide participants with information about their teammates’ strength, freeze frames with text were presented for 20 s at the beginning and in the middle of each block. In this text, participants in the explicit groups of both distributions (67% and 83%, respectively) were told that the weak teammate mostly loses and the strong teammate mostly wins the 1-on-1 situations. Both teammates could be identified by their jersey numbers. In contrast, the participants in the self-generated groups were just informed that their teammates will either win 1-on-1 situations by making a foul according to the rules or lose by allowing the attacker to throw at the goal. Hence, this information included no hints about the experimentally induced strength patterns in the 1-on-1 situations. As these freeze-frame messages were repeated twice per block, participants received this information twelve times over the whole experiment.

During the experimental blocks, participants acted as the central defender in the displayed 3 on 3 defensive situations. They were instructed to move as they would in a real-world environment, with the ultimate objective to prevent goals. After the last block of the experiment, an explicit-knowledge test was conducted and a background questionnaire was completed (including age, practice experience, competition experience, average playing hours per week, and average games per season). Participants were finally thanked and debriefed on the objectives of the study.

### Dependent measures

#### Strength-dependent positional difference

As a sensitive behavioral measure of the effects of contextual knowledge, participants’ individual defensive movements in the experimental trials were analyzed in relation to their teammate’s success [21, 32]. Specifically, if a teammate is going to lose a 1-on-1 situation, the participant should move closer to support this teammate, such that the participant could tackle the opposing attacker. In contrast, if a teammate is going to win a 1-on-1 situation, the participant should maintain her central position and allow the teammate to successfully foul the attacker themself. Importantly, the suitability of a participant’s behavioral responses was assessed in relation to the actual situation; that means, regardless of whether it concerned the strong or a weak teammate.

In more detail, two time points were specified in the video recordings; namely, ball contact and ball release (cf. Fig 1). Ball contact was identified as the moment the offensive player catches the ball before the 1-on-1 situation occurred. Ball release was identified as either the last moment the offensive player touched the ball when throwing or, in cases of successful defensive actions of the teammate, the last moment the offensive player could have passed the ball to the middle back (i.e., the participants’ direct opponent) before being fouled by the defender. For both time points, the respective video frame was independently determined by three handball-experienced raters (ball contact: *M*_difference_ = 0.82 frames, IRR = .972; ball release: *M*_difference_ = 4.53 frames, IRR = .967) and the median frame was taken as the decisive frame.

At ball release, the participant’s current position on the floor was calculated as the mean x-value (left/right dimension) of the four head and back markers. In order to relate the lab-referenced positional data to a more meaningful origin, the x-data for all experimental trials of all participants was centered at the overall mean position at ball contact in the 32 trials of the first block. From the CAVE’s left side, the new origin was located at 2.82 m (cf. dotted white line in Fig 1). From this frame of reference, the position of each participant at ball release was determined for all experimental trials in the early and late acquisition phases of the experiment. Subsequently, the x-data for defensive movements to the left were inverted in such a way as if all defensive movements had been performed to the right, which facilitates the aggregation of a comprehensive dependent variable. In the subsequent analyses, only the data of the head markers were used for calculating a dependent measure for defensive behavior.

In the early and late acquisition phases of the experiment, a total of 82 (= 1.6%) missing values were identified for the head markers (pattern-consistent trials: 53 = 1.5%, pattern-inconsistent trials: 29 = 1.7%). However, in 58 of these cases (pattern-consistent trials: 35, pattern-inconsistent trials: 23), the data for the back markers was available to estimate the gapped position by adding the averaged difference between the head and back markers of the respective participant in trials of the specific phase (i.e., early or late) in the specific pattern condition (i.e., consistent or inconsistent) to the value of the back markers. Data from the back markers was unavailable from the remaining 24 missing values from the head markers (pattern-consistent trials: 18, pattern-inconsistent trials: 6). Therefore, the head positions were estimated as the averaged value of existing head data of the respective participant in trials of the specific phase (i.e., early or late) in the specific pattern condition (i.e., consistent or inconsistent).

On this basis, the dependent variable of strength-dependent positional difference was finally calculated as the difference between the averaged x-values for all scenes in which a teammate lost the 1-on-1 situation and the averaged x-values for all scenes in which a teammate won the situation, respectively–again, regardless of the teammates’ general defense strengths. Consequently, higher values in the positional-difference variable indicate that participants moved farther away from the central position; or in other words, closer to the teammate who required help due to a weak defensive action. If having generated or acquired applicable contextual knowledge, participants could expect the strong teammate to require less help than the weak teammate. Therefore, the resulting strength-dependent positional difference can be recognized as a valid measure for participants’ behavioral control based on contextual knowledge.

#### Information gain

The calculation of the individual information gain of explicitly provided contextual knowledge in relation to expertise (i.e., expert vs. near-expert) as well as information certainty (i.e., 67% vs. 83%) was not possible based on the observed data as participants partook the experiment only in one of the acquisition conditions. To relate the data obtained to the theoretical framework, we therefore had to adopt an alternative statistical approach. For this purpose, a dataset with 2 x 36 estimated information gains was generated out of the 2 x 72 original values (i.e., positional difference of each participant for the early and late acquisition phase). Hereby, issues in terms of data quality, statistical validity and practical feasibility had to be considered. To this end, we introduced a permutation-based approach that (i) does not require strong assumptions about the distribution of the data or the relationships between variables, (ii) represents variations of the original data so that plausible estimates of variability–due to chance as well–can be created, (iii) prevents any structural biases in the estimated dataset, and (iv) ‘virtually’ expands the small sample to produce more robust results [35–37]. Specifically, for each participant of one of the four explicit groups, the strength-dependent positional difference was subtracted ninefold from the positional difference of all participants of the respective self-generated group, separately for the early and late acquisition phase of the experiment. For instance, the early acquisition phase value of participant 1 (i.e., expert, 87% certainty, and explicit knowledge provision) was subtracted from the values of participants 10–18 (i.e., experts, 87% certainty, and self-generated knowledge) so that nine values for an information gain resulted. In doing so, the permutation-based approach allowed to generically produce an estimated data set from the original values by repeatedly compare one value with the values’ distribution of the other condition. In total, this resulted in 324 values (4 explicit groups x 9 participants per group x 9 permutations per participant). Subsequently, for each explicit group, nine cases were calculated as the mean of the nine permutations so that a total of 36 values (4 explicit groups x 9 participants per group) resulted which were followingly used for the analysis of the four groups. Consequently, a positive information-gain value (e.g., 0.3m) would indicate that participants in the explicit groups had a greater positional difference compared to the participants in the respective self-generated groups. More specifically, a greater positive value indicates that participants moved closer to their teammate when help was required and stayed more central when the teammate managed to stop the attacker with a foul.

#### Pattern detection

The degree to which the participants became consciously aware of the experimentally induced strength patterns was inferred from the responses given in an explicit-knowledge test. The test consisted of two questions and was conducted at the end of the experimental session. As an indirect measure of pattern detection, the first item was an open question that asked whether the participants had noticed any specific behavioral patterns of their teammates in particular situations. The responses were assessed with respect to the participants’ awareness of the difference in their teammates’ defensive strengths and rated by three independent raters on a 4-point scale from *no detection at all* (= 1) to *completely detected* (= 4) (IRR = .883). The second question directly probed the participants’ awareness of their teammates’ defensive strength; asking if participants detected (i.e., 67% or 83%) on a 4-point scale from *did not know at all* (= 1) to *knew for sure* (= 4). Subsequently, pattern detection for the eight groups was calculated as the average score of the indirect and direct questions, respectively.

### Data analysis

Statistical tests were conducted using SPSS 28.0 (SPSS Inc., Chicago, IL, USA). A two-way ANOVA on expertise (2) x acquisition phase (2), with repeated measures on the last factor, was used to assess expertise differences and behavioral learning for the dependent variable of strength-dependent positional difference. A three-way ANOVA on expertise (2) x information certainty (2) x acquisition phase (2), with repeated measures on the last factor, was conducted in regards to the main research question on information gain of explicitly provided contextual knowledge. Furthermore, three-way ANOVAs on expertise (2) x information certainty (2) x acquisition condition (2) were conducted to assess the degree of pattern detection, as well as potential effects of demographic characteristics (as reported above). Greenhouse-Geisser corrections were applied in cases of violations of sphericity. Partial eta square (η_p_^2^) and confidence intervals (95% CI) were calculated as measures of effect size. The alpha level for all statistical tests was set *a priori* at *α* = .05. Additionally, non-significant statistical tests of core hypotheses were supported by Bayesian analyses using MorePower 6.0.4 so that the distinction between null and indeterminate findings was possible by directly estimating the posterior probability distribution of the hypothesized effect [33].

### Transparency and openness

The raw data have been made publicly available at the Bern Open Repository and Information System (BORIS) and can be accessed at https://boris.unibe.ch/id/eprint/185108" \t "_top.

## Results

For the sake of clarity, only the relevant ANOVA effects are illustrated in figures below. Descriptive statistics for positional difference, information gain, and pattern detection can be found in S2–S4 Tables.

### Strength-dependent positional difference

**Fig 2:**
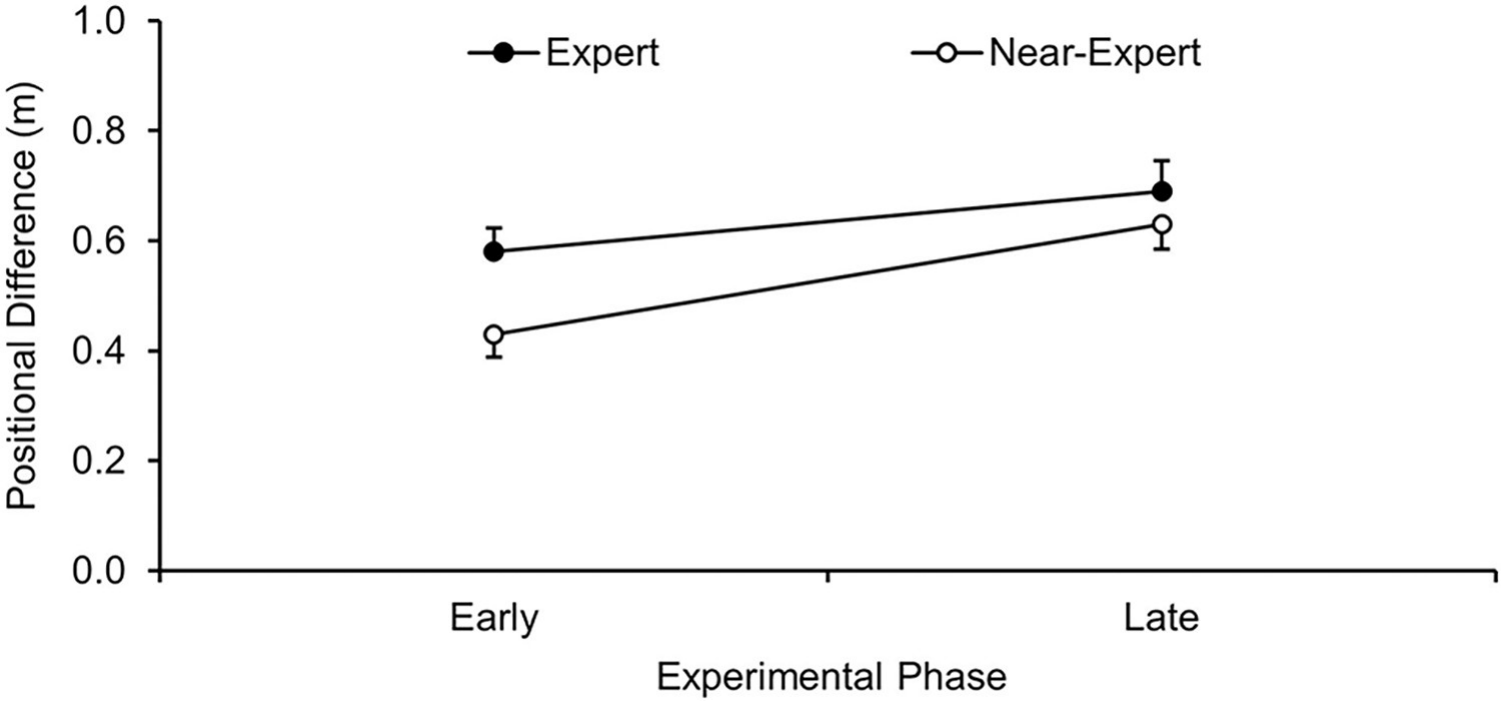
Strength-dependent positional difference (M and SE) as a function of expertise (expert, near-expert) and acquisition phase (early, late).

To check whether contextual knowledge influenced behavioral control in relation to expertise and acquisition phase, a two-way ANOVA expertise (2) x acquisition phase (2), with repeated measures on the last factor, was conducted for strength-dependent positional difference. The analysis revealed a significant main effect for expertise, *F*(1, 70) = 3.14, *p* = .041, η_p_^2^ = .04, and for phase, *F*(1, 70) = 26.66, *p* < .001, η_p_^2^ = .28, but an indeterminate interaction, *F*(1, 70) = 2.66, *p* = .107, η_p_^2^ = .04. As depicted in Fig 2, expert players generally moved closer to their teammate in situations which required help and stayed more central when their teammate managed to stop the attacker with a foul (*M*_*difference*_ = 0.11 m, 95% CI = [-0.01; 0.22]). Furthermore, both expertise groups improved their strength-dependent positional difference from the early to the late acquisition phase of the experiment (*M*_*difference*_ = 0.16 m, 95% CI = [0.10; 0.22]).

### Information gain

**Fig 3:**
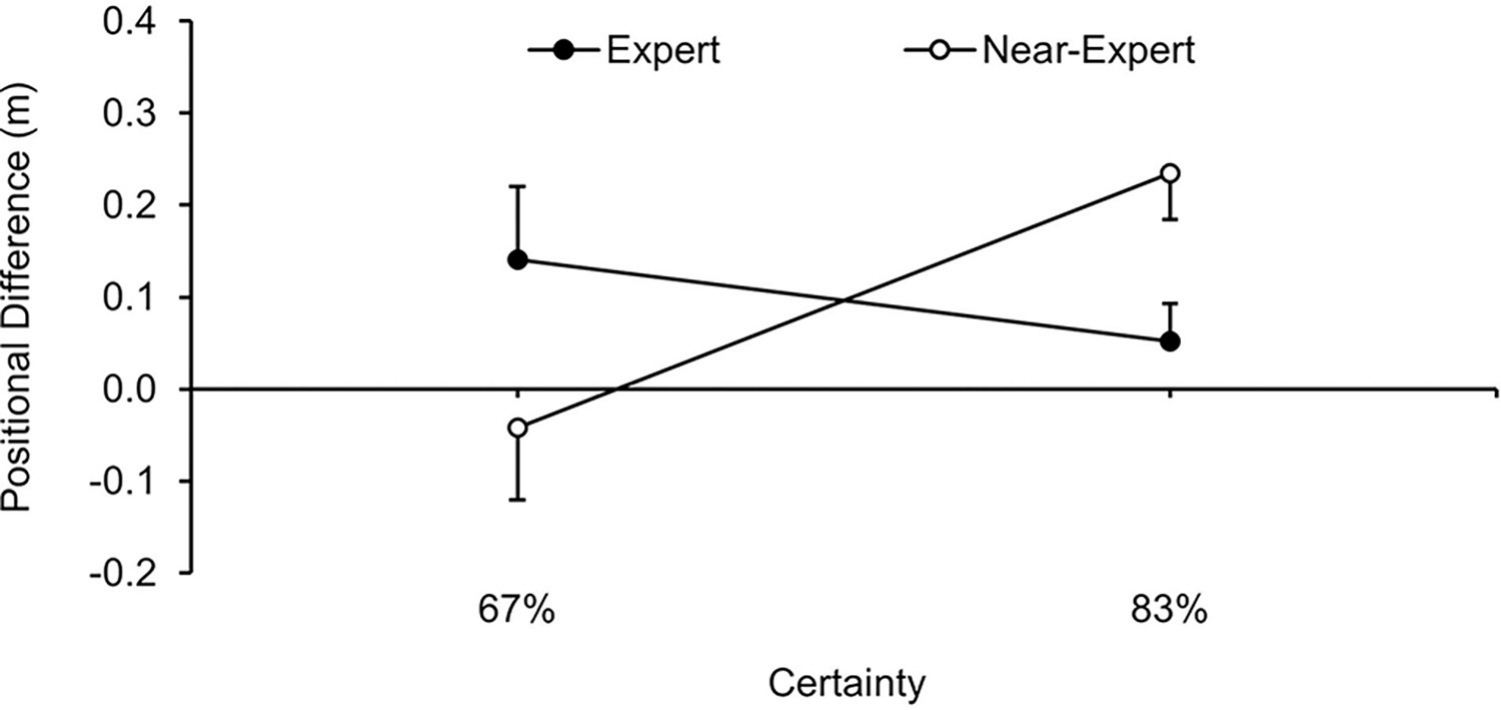
Information gain of explicitly provided information (M and SE) for the 67% and the 83% certainty groups as a function of expertise (expert vs. near-expert).

To assess the benefits of the explicit provision compared to the self-generated acquisition of contextual information as a function of expertise, information certainty and acquisition phase (i.e., the available time for self-generation of knowledge), the information gain for each group was calculated by the permutation procedure described in the methods section on the basis of all values of the participants of the explicit and self-generated information groups. A three-way ANOVA expertise (2) x information certainty (2) x acquisition phase (2), with repeated measures on the last factor, indicated indeterminate main effects on information gain, *F*s(1, 32) ≤ 1.31, *p*s ≥ .261, η_p_^2^s ≤ .04. Apart from a significant two-way interaction for expertise x information certainty, *F*(1, 32) = 5.01, *p* = .016, η_p_^2^ = .14, no other interaction was found to be significant, *F*s(1, 32) ≤ 2.56, *p*s ≥ .120, η_p_^2^s ≤ .07. A Bayesian analysis of the three-way interaction provided substantial evidence for the non-significant result (ΔBIC = 2.265, BF = 3.103, *p*_BIC_(H_0_|D) = .756, and *p*_BIC_(H_1_|D) = .244). The two-way interaction effect for expertise x information certainty–which is not overlaid by any acquisition-phase differences–is depicted in Fig 3. Post hoc comparisons revealed no significant certainty effect for experts, *F*(1, 34) = 1.01, *p* = .322, η_p_^2^ = .03, which was confirmed by substantial evidence of the Bayesian analysis (ΔBIC = 2.465, BF = 3.430, *p*_BIC_(H_0_|D) = .774, and *p*_BIC_(H_1_|D) = .226). Contrastingly, the comparisons showed a significant certainty effect for near-experts, *F*(1, 34) = 8.83, *p* = .005, η_p_^2^ = .21, meaning that the information gain increased with information of higher certainty (Δ_information gain_ = 0.28 m, 95% CI = [0.09; 0.47]).

### Pattern detection

**Fig 4:**
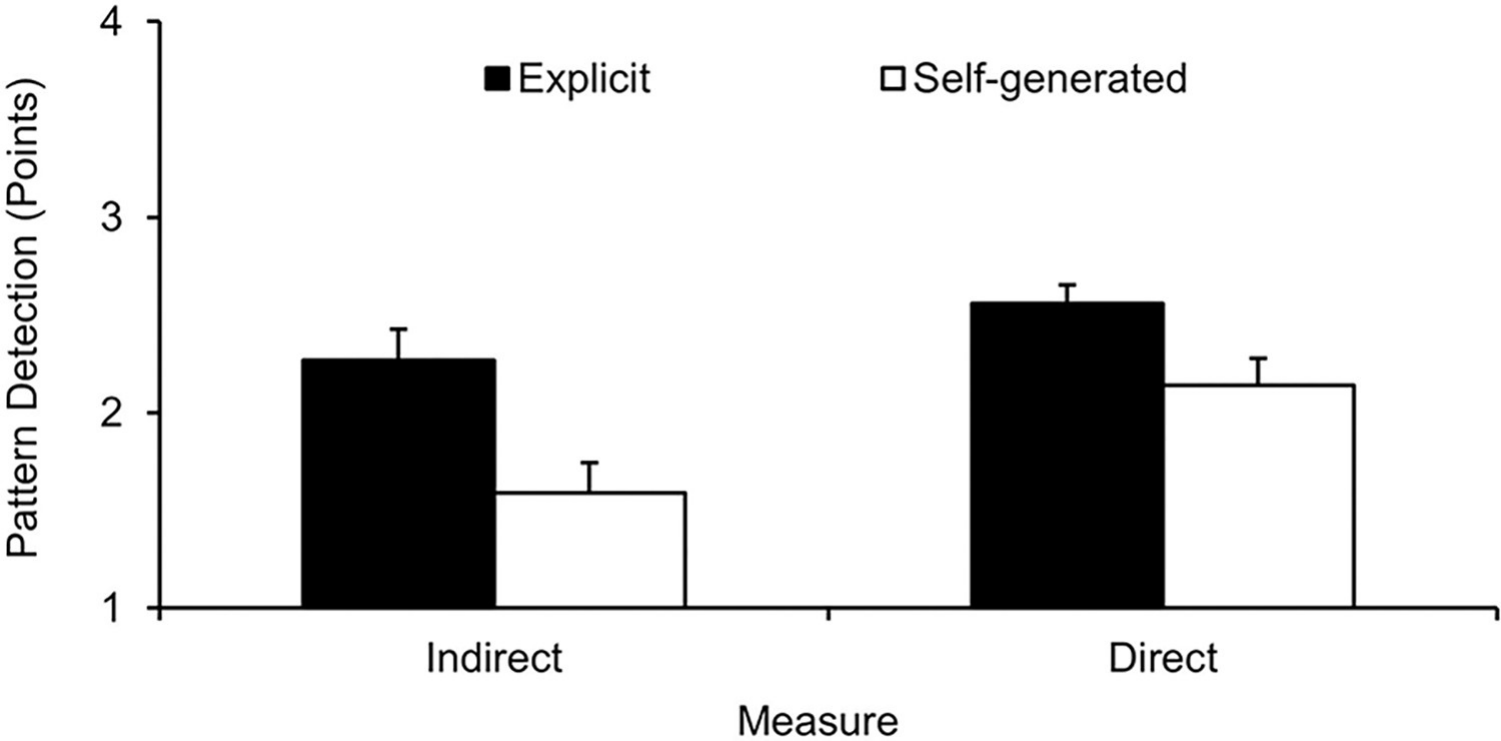
Pattern detection (M and SE) for indirect and direct measure as a function of acquisition condition (self-generated vs. explicit).

To examine players’ ability to detect their teammates’ defensive strength patterns, the indirect and direct knowledge scores were analyzed. For indirect pattern measure, a three-way ANOVA expertise (2) x information certainty (2) x acquisition condition (2) revealed a significant main effect for acquisition condition, *F*(1, 64) = 9.55, *p* = .003, η_p_^2^ = .13, indeterminate main effects, *F*s(1, 64) ≤ 3.01, *p*s ≥ .087, η_p_^2^s ≤ .05, and indeterminate interaction effects, *F*s(1, 64) ≤ 0.79, *p*s ≥ .377, η_p_^2^s ≤ .01. Particularly, a Bayesian analysis of the main factor certainty provided only anecdotal evidence for the non-significant result (ΔBIC = 1.569, BF = 2.191, *p*_BIC_(H_0_|D) = .687, and *p*_BIC_(H_1_|D) = .313). Similarly, a three-way ANOVA expertise (2) x information certainty (2) x acquisition condition (2) on direct pattern measure showed a significant main effect for acquisition condition, *F*(1, 64) = 6.08, *p* = .016, η_p_^2^ = .09, but no other main effects, *F*s(1, 64) ≤ 2.43, *p*s ≥ .624, η_p_^2^s < .01, and indeterminate interaction effects, *F*s(1, 64) ≤ 3.27, *p*s ≥ .075, η_p_^2^s ≤ .05. The non-significant effects of the main factors were supported by a Bayesian analysis that provided substantial evidence for these results (expertise: ΔBIC = 4.016, BF = 7.445, *p*_BIC_(H_0_|D) = .882, and *p*_BIC_(H_1_|D) = .118; information certainty: ΔBIC = 4.004, BF = 7.403, *p*_BIC_(H_0_|D) = .881, and *p*_BIC_(H_1_|D) = .119). Post hoc pairwise comparisons revealed a significant effect of acquisition condition for the indirect pattern measure, *F*(1, 70) = 9.42, *p* = .002, η_p_^2^ = .12, as well as for the direct pattern measure, *F*(1, 70) = 6.22, *p* = .008, η_p_^2^ = .08. As depicted in Fig 4, independent of expertise and information certainty, the explicit groups were better able to detect the experimentally induced pattern in both measures (indirect measure: *M*_difference_ = 0.68, 95% CI = [0.24; 1.11], direct measure: *M*_difference_ = 0.42, 95% CI = [0.08; 0.75]).

## Discussion

Behavioral control in complex, real-world settings–particularly in the context of decision-making in sport games–is characterized by a considerable amount of uncertainty. To reduce uncertainties, decision-making comes down to optimally using the available information by merging incoming sensory inputs with prior contextual knowledge. Contextual knowledge is typically corrupted by uncertainty itself and it can be built on with explicitly provided information or be self-generated by extracting situational probabilities from one’s own experiences. Thus, the question arises to what extent the information gain from the explicit provision of contextual information affects behavioral control. Therefore, the present experiment investigated the gain of explicitly provided contextual information in a complex handball-defense task as a function of expertise level, the (un)certainty of information and the acquisition phase, thereby using the field positioning of players as a reference. Additionally, we examined players’ ability to verbalize the experimentally induced strength patterns in the presented scenarios.

In summary, the results of the players’ positional difference show that, unsurprisingly, all players improved from the early to the late acquisition phase of the experiment (see Fig 2). This finding is perfectly in line with previous research regarding the use of contextual knowledge for anticipatory behavior in general [10, 16–18, 22–25] as well as specifically in handball defense [21, 32]. Similarly, the generally superior performance of the experts compared to their near-experts counterparts confirms recent findings [10, 16, 17, 19, 20]. In this regard, the performance difference already detected in the early acquisition phase should be attributed to a more effective use of highly reliable kinematic information of the teammates and opponents to anticipate the upcoming action [38]. Notably, however, the available visual cues cannot further explain performance or performance enhancement effects as the virtual-reality setup allowed us to keep them perfectly constant over the entire experiment so that revealed effects can be reliably assigned to the acquired contextual knowledge.

For the indirect and direct knowledge tests conducted at the end of the experiment, it was found that players who received the explicit information about the teammates’ strength were generally better able to explicitly verbalize the experimentally induced pattern, as expected based on previous research ([31]; see Fig 4). However, our prediction that the higher-certainty groups would outperform the lower-certainty groups in the knowledge tests [32] was not confirmed by the present findings. Notably, neither a general expertise effect was revealed nor did the expertise level affect any other variable (i.e., information certainty and acquisition condition). These results thus imply that the ability to explicitly verbalize a sport-specific pattern is simply related to whether the information was explicitly provided or not. Consequently, examining this kind of explicit knowledge seems of no additional value due to the limited contribution for the explanation of differences in behavioral control.

In terms of the information gain as the most relevant dependent variable of the present study, our main finding regards the predicted significant interaction of expertise and (un)certainty of provided information (see Fig 3). Whilst on the one hand, experts slightly benefited from the explicit provision of contextual information–independent of the information certainty (see positive values in Fig 3)–, the certainty of provided information remarkably affected the gain in near-experts. Due to the considerable effect size, this finding highlights the practical relevance. A clear beneficial effect was found in near-experts with higher certainty information–which, in turn, corresponds with recent research showing that female soccer players benefited more from explicit contextual knowledge when the information was of high reliability [8]. Contrastingly, explicitly provided contextual information with lower certainty had a negative impact on behavioral control of near-experts (see negative values in Fig 3). It seems reasonable to attribute this detrimental effect mainly to impaired decisions in pattern-inconsistent situations; that being, in the present study, in situations when either the strong teammate lost or the weak teammate won against the attacker. Interestingly, this interpretation is also supported by the partially observable Markov decision process simulations of Harris et al. [5]. For experts, in contrast, the higher uncertainty caused by a larger percentage of pattern-inconsistent situations apparently did not affect their behavioral control negatively (i.e., 67% expert group in Fig 3). For experts, the explicit provision of the contextual information was thus generally beneficial. This result contradicts previous findings regarding the congruency effect in which skilled players showed a performance decrease in incongruent trials for self-generated as well explicitly provided contextual knowledge [19, 20, 24, 25].

However, further predictions could not be confirmed by the present study. This especially applies to the fact that the data for lower certainty does neither indicate a general superior information gain for near-experts nor a harmful effect for experts, as expected on the basis of the reported previous research on motor learning [32]. Furthermore, the prediction of a generally more pronounced information gain for the higher-certainty groups [30, 32] was only confirmed for the near-experts but not for the experts. Moreover, these results are not in line with the classic Fitts and Posner model, which implies that players of lower expertise principally rely on explicit knowledge and players of higher expertise on implicit knowledge [39], nor the assumption that learners with lower-certainty information would benefit more from implicit or self-generated acquisition than from explicit instructions [15, 26, 27, 30]. And finally, our findings did not show that learners generally suffer from explicit-information provision in complex tasks due to high attentional demands (i.e., updating and integrating contextual and kinematic information) as reported in other research [4, 24, 28]. We suggest that this discrepancy is not explained by the contextual information itself as in the current study stable information was used as well; we rather argue that the explicit instruction might have affected the task load differently. While detrimental effects were found when contextual information was provided in the form of probabilistic percentages forcing the athletes to calculate exact probabilistic rules [24], the handball players were simply provided with the information that the weak teammate mostly loses and the strong teammate mostly wins the 1-on-1 situations. Thus, future research should examine the effect of different ways to explicitly provide athletes with contextual information.

Mixed conforming results must also be reported when comparing the present findings on a more fine-grained level with the model estimations calculated by Magnaguagno et al. [32], in which effects of the acquisition process are taken into account as well (see S2–S4 Tables). In this regard, a rather satisfying model fit–at least by tendency–can be stated for higher-certainty conditions since both expertise groups benefited to a particular extent–near-experts more pronounced than experts–from the explicit provision of contextual information in the early acquisition phase. This effect is more preserved for near-experts than for experts in the late acquisition phase. The same holds for less certain conditions in the early acquisition phase; for which only marginal performance differences were predicted between self-generated and explicitly provided contextual information. However, for the late acquisition phase, when a knowledge base can be expected to be sufficiently established, the present data contradicted the model. Explicit information provision actually affected experts’ behavior positively and impaired near-experts’ performance. Summing up, our current findings imply that explicitly providing players with contextual information is unlikely to harm the players’ behavioral control, and the expectable information gain depends on various factors, such as expertise level and the certainty of the provided information. Thus, this discrepancy calls for further discussions that, in our eyes, should be related to the following three shortcomings of the present study.

(i) An obvious limitation of the current study regards the calculation of the information-gain variable from differences between groups with and without the explicit provision of contextual information. With this procedure, it is apparently not possible to analyze the ‘true’ information gain on an individual level. Thus, in future research, participants should be tested under both conditions. In doing so, the decision of whether to provide learners with explicit contextual information–or rather to refrain from information provision–would be grounded on individual prerequisites rather than the affiliation to a specific group.

(ii) As a further limitation, we examined adult female handball players in the present study whilst youth male handball players were recruited in the Magnaguagno et al. [32] experiment and, due to availability, both studies presented adult male models in the experimental videos. The observed discrepancy between the obtained results might thus also be ascribed to those differences in gender, expertise level and the fit between presented players and one’s own gender and age, respectively. Furthermore, this means that the study’s scope is limited to this particular population and presentation conditions such that transfers of the effects into other contexts should only be made cautiously.

(iii) Consequently, there seems to be a need to take such individual factors into account; including the consideration of individual gain values as well as aspects of gender, age and fit of presented players with participants. In the context of the self-generated vs. explicit learning distinction at hand, these personal attributes seem to be particularly relevant to personality factors that are known to relate to implicit and explicit learning (e.g., visuo-spatial working memory). Such factors might form crucial mediating variables to thoroughly explain information-provision effects.

Taken together, it can be concluded that sport game players should generally be provided with contextual information by their coaches as the provision is overall not detrimental. However, to improve the provision-related information gain, moderating factors should be considered. Particularly, skilled players benefit when the information is uncertain and providing less-skilled players is advantageous in situations under high certainty. In this context, it seems reasonable that coach instructions have to be limited to the most game relevant contextual information–increasing the value of targeted game analysis–and in a core-grained manner so that task load does not compromise the information gain. Additionally, a frequent repetition of the information provision could be advisable, meaning that coaches should convey the information to their players several times. Different forms of instruction (e.g., nonverbal signs) could also be exploited as there is usually no time for detailed instructions during a game. While we assume that the obtained gain of provided contextual information is transferable to other sport games, an expansion of the application on decision-making domains outside of the sport game context would be of considerable interest. In this regard, it seems plausible that similar effects only occur for decisions which have to be made under high time pressure without the availability of reliable kinematic information and where a complex behavioral control is required.

Finally, the points (i)–(iii) support the pursuit of a more differential approach to study the effects of explicit information provision vs. self-generation of contextual knowledge in complex sensorimotor behavior. In this regard, the present study–which demonstrated that information gain depends on the interaction of expertise level and information certainty–may be seen as a reasonable step into this direction. The effects of further variables should be targeted in future research.

## Supporting information

S1 Table Overview of the experimental design with the number of scenes per subcategory in brackets. (PDF)

S2 Table Strength-dependent positional difference (M and SE) as a function of expertise (Experts, near-expert), certainty (67%, 83%), acquisition phase (Early, late) and acquisition condition (Self-generated, explicit). (PDF)

S3 Table Information gain (M and SE) as a function of expertise (Experts, near-expert), information certainty (67%, 83%) and acquisition phase (Early, late). (PDF)

S4 Table Pattern detection (M and SE) for the indirect and direct measure as a function of expertise (Experts, near-expert), information certainty (67%, 83%5/6) and acquisition condition (Self-generated, explicit). (PDF)
