# Supplementary material for: The information gain of explicitly provided over self-generated contextual knowledge for behavioral control
Source: PLoS One. 2025 Feb 7;20(2):e0318994. doi: 10.1371/journal.pone.0318994 (PMC11805413; doi:10.1371/journal.pone.0318994)
Supplement: S1 Table — (PDF) [file pone.0318994.s001.pdf]

S1 Table. Overview of the Experimental Design with the Number of Scenes per Subcategory in Brackets.

		Experimental scenes (84)				Additional scenes (48)
		Weak (42)<br>67% or 83% losing		Strong (42)<br>67% or 83% winning		
		Left (21)	Right (21)	Left (21)	Right (21)	Left/Right
Early (64)	Block 1 (32)	6	6	6	6	8
	Block 2 (32)	6	6	6	6	8
	Block 3 (32)	6	6	6	6	8
	Block 4 (32)	6	6	6	6	8
Late (64)	Block 5 (32)	6	6	6	6	8
	Block 6 (32)	6	6	6	6	8
