# Supplementary material for: The information gain of explicitly provided over self-generated contextual knowledge for behavioral control
Source: PLoS One. 2025 Feb 7;20(2):e0318994. doi: 10.1371/journal.pone.0318994 (PMC11805413; doi:10.1371/journal.pone.0318994)
Supplement: S2 Table — (PDF) [file pone.0318994.s002.pdf]

**S2 Table. Strength-Dependent Positional Difference (M and SE) as a Function of Expertise (Experts, Near-Expert), Certainty (67%, 83%), Acquisition Phase (Early, Late) and Acquisition Condition (Self-generated, Explicit).**

| | | Self-generated | | Explicit | |
|---|---|---|---|---|---|
| | | Early | Late | Early | Late |
| Experts | 67% | 0.55 (0.06) | 0.55 (0.08) | 0.59 (0.07) | 0.78 (0.07) |
| | 83% | 0.55 (0.05) | 0.70 (0.04) | 0.64 (0.08) | 0.72 (0.07) |
| Near-Experts | 67% | 0.44 (0.07) | 0.64 (0.06) | 0.41 (0.06) | 0.58 (0.06) |
| | 83% | 0.31 (0.06) | 0.55 (0.05) | 0.56 (0.07) | 0.76 (0.07) |
