# Supplementary material for: The information gain of explicitly provided over self-generated contextual knowledge for behavioral control
Source: PLoS One. 2025 Feb 7;20(2):e0318994. doi: 10.1371/journal.pone.0318994 (PMC11805413; doi:10.1371/journal.pone.0318994)
Supplement: S3 Table — (PDF) [file pone.0318994.s003.pdf]

S3 Table. Information Gain (M and SE) as a Function of Expertise (Experts, Near-Expert), Information Certainty (67%, 83%) and Acquisition Phase (Early, Late).

		Early	Late
Experts	67%	0.05 (0.07)	0.24 (0.14)
	83%	0.08 (0.06)	0.02 (0.05)
Near-Experts	67%	-0.03 (0.09)	-0.06 (0.14)
	83%	0.26 (0.08)	0.21 (0.06)
