# Supplementary material for: The information gain of explicitly provided over self-generated contextual knowledge for behavioral control
Source: PLoS One. 2025 Feb 7;20(2):e0318994. doi: 10.1371/journal.pone.0318994 (PMC11805413; doi:10.1371/journal.pone.0318994)
Supplement: S4 Table — (PDF) [file pone.0318994.s004.pdf]

S4 Table. Pattern Detection (M and SE) for the Indirect and Direct Measure as a Function of Expertise (Experts, Near-Expert), Information Certainty (67%, 83%5/6) and Acquisition Condition (Self-generated, Explicit).

		Indirect Measure		Direct Measure	
		Self-generated	Explicit	Self-generated	Explicit
Experts	67%	1.63 (0.33)	2.44 (0.32)	2.22 (0.32)	2.44 (0.18)
	83%	1.74 (0.35)	2.67 (0.29)	2.00 (0.29)	2.89 (0.11)
Near-Experts	67%	1.22 (0.08)	1.74 (0.26)	2.00 (0.29)	2.56 (0.18)
	83%	1.78 (0.39)	2.22 (0.34)	2.33 (0.24)	2.33 (0.24)
